# Highly Efficient Photocatalytic Degradation of Bisphenol A Under UV–Visible Light Irradiation Using Au/Zn_3_In_2_S_6_ Schottky Junction Photocatalyst

**DOI:** 10.3390/ijms27020705

**Published:** 2026-01-10

**Authors:** Di Chen, Aoyun Meng, Zhen Li, Jinfeng Zhang

**Affiliations:** 1 Key Laboratory of Molecular Biology on Infectious Disease, Ministry of Education, Chongqing Medical University, Chongqing 400016, China; 19845414879@163.com; 2 College of Food Science and Engineering, Anhui Science and Technology University, Chuzhou 239000, China; yjs2023261@ahstu.edu.cn; 3 Anhui Province Key Laboratory of Functional Agriculture and Functional Food, Anhui Science and Technology University, Chuzhou 239000, China; 4 College of Physics and Electronic Engineering, Huaibei Normal University, Huaibei 235000, China

**Keywords:** photocatalytic, Schottky junction, bisphenol A, environmental remediation

## Abstract

Designing and constructing heterojunctions has emerged as a pivotal strategy for improving the photocatalytic efficiency of semiconductors. In this study, we report the controlled synthesis of an Au/Zn_3_In_2_S_6_ Schottky junction through a combination of hydrothermal and in situ photodeposition methods. The structural, morphological, and photoelectrochemical properties of the catalyst were meticulously characterized using a suite of techniques including X-ray diffraction (XRD), scanning electron microscopy (SEM), transmission electron microscopy (TEM), diffuse reflectance spectroscopy (DRS), X-ray photoelectron spectroscopy (XPS), photoelectrochemical (PEC) measurements, and electron spin resonance (ESR) spectroscopy. The optimized 3% Au/Zn_3_In_2_S_6_ composite exhibited a remarkable enhancement in both photocatalytic activity and stability, achieving a 90.4% removal of bisphenol A (BPA) under UV–visible light irradiation within 100 min. The corresponding first-order reaction rate constant was approximately 1.366 h^−1^, nearly 4.37 times greater than that of the pristine Zn_3_In_2_S_6_. This substantial improvement can be attributed to several key factors, including increased BPA adsorption, enhanced light absorption, and the efficient charge separation facilitated by the Au/Zn_3_In_2_S_6_ heterojunction. Photogenerated holes, superoxide radicals, and hydroxyl radicals were identified as the primary reactive species responsible for the BPA degradation. This work highlights the potential of metal-modified semiconductors for advanced photocatalytic applications, offering insights into the design of highly efficient materials for environmental remediation.

## 1. Introduction

In recent decades, growing concerns over environmental issues, particularly water pollution caused by pharmaceutical and industrial wastewater [1,2,3,4,5,6], which is closely linked to the occurrence of various diseases [7], have driven the demand for the development of new environmental remediation technologies [8,9,10,11,12,13]. Among various organic pollutants, bisphenol A (BPA), an endocrine-disrupting environmental hormone, stands out as one of the most difficult pollutants to treat due to its colorless nature, widespread presence in natural water, and resistance to decomposition [14,15,16,17,18,19,20]. As a result, BPA has attracted considerable attention in recent years. Among the various methods for BPA removal, semiconductor photocatalysis has emerged as a promising solution due to its environmentally friendly, solar-light-driven process that offers safety and sustainability [21,22,23,24,25,26,27]. Therefore, the exploration of semiconductor photocatalysts for efficient BPA degradation has become a major research focus [28,29,30,31,32,33,34].

Building upon the success of previous photocatalytic materials, Zn_3_In_2_S_6_ (ZIS6), an n-type semiconductor with visible-light responsiveness, has garnered significant attention in recent years as a potential photocatalyst due to its advantages, such as easy preparation, low-dimensional structure, tunable bandgap, and the possibility for surface modifications [35,36,37,38,39,40]. ZIS6 has already shown promise in various applications, including photocatalytic hydrogen production, hydrogen peroxide synthesis, selective oxidation-coupled hydrogen production, CO_2_ reduction, and pollutant degradation. However, the photocatalytic performance of pristine ZIS6 is often limited by the rapid recombination of photogenerated electrons and holes [41,42,43]. To enhance its photocatalytic activity, numerous strategies have been proposed, with the construction of heterojunctions being one of the most effective approaches for promoting the separation and migration of photogenerated charges [44,45,46,47,48,49,50,51,52].

Recently, Li et al. reported a strategy for tailoring redox active sites with dual-interfacial electric fields to enhance photocatalytic biomass valorization and hydrogen production. This strategy significantly improved the overall photocatalytic performance, demonstrating its versatility in various photocatalytic processes [53]. On the other hand, the study on Au/Cu-Zn_3_In_2_S_6_ decoupling of carrier pathways through bulk hole trapping and surface hot electron accumulation demonstrates the advantages of this approach in enhancing photocatalytic hydrogen peroxide production. The Au/Cu-Zn_3_In_2_S_6_ system, fabricated via a unique heterostructure design, exhibited significantly enhanced photocatalytic activity, showcasing its potential in energy and environmental applications [54]. Clearly, the design and construction of heterojunctions remain an effective strategy for enhancing photocatalytic efficiency [55,56,57,58,59].

In this work, we first prepared the Au/Zn_3_In_2_S_6_ (Au/ZIS6) heterojunction and applied it for the first time to BPA degradation. A Schottky junction was formed at the interface between ZIS6 and Au due to the difference in Fermi levels, which significantly inhibited charge recombination within ZIS6. The incorporation of gold was chosen due to its ability to enhance light absorption through surface plasmon resonance, promote efficient charge separation, and improve the adsorption of BPA, all of which significantly boost the photocatalytic activity of ZIS6. Au modification facilitated BPA adsorption and enhanced light absorption. The enhancement in BPA adsorption can be attributed to the strong interaction between the Au nanoparticles and BPA molecules, facilitated by the localized surface plasmon resonance (LSPR) effect. This effect increases the surface charge density and improves the catalyst’s interaction with BPA, leading to higher adsorption efficiency. The photocatalyst was comprehensively characterized using a range of techniques, including X-ray diffraction (XRD), scanning electron microscopy (SEM), transmission electron microscopy (TEM), diffuse reflectance spectroscopy (DRS), X-ray photoelectron spectroscopy (XPS), and photoelectrochemical (PEC) measurements. These characterizations confirmed the successful formation of the Au/ZIS6 heterojunction and revealed insights into its improved photocatalytic activity. The optimized 3% Au/ZIS6 composite exhibited significantly enhanced photocatalytic activity and stability for BPA degradation, achieving 90.4% BPA removal under UV–visible light irradiation within 100 min. The first-order reaction rate constant was approximately 1.366 h^−1^, approximately 4.37 times higher than that of the pristine ZIS6. This improvement is attributed to the enhanced BPA adsorption, increased light absorption, and efficient charge separation facilitated by the Au/ZIS6 heterojunction. Photogenerated holes, superoxide radicals, and hydroxyl radicals were identified as the primary reactive species responsible for the degradation of BPA.

## 2. Results and Discussion

### 2.1. Material Preparation Flowchart

**Figure 1:**
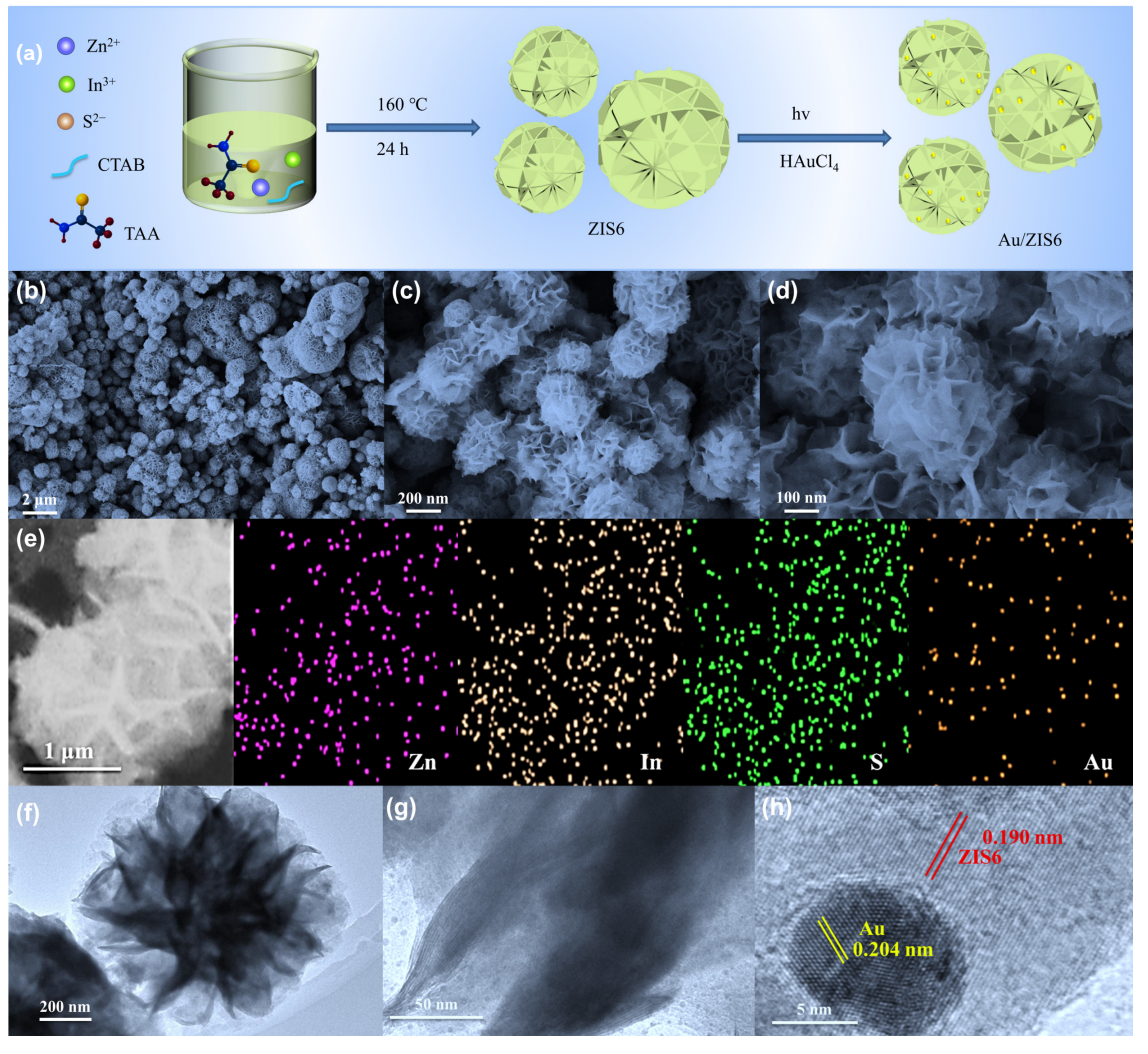
(**a**) The schematic diagram of the synthesis of Au/ZIS6. (**b**–**d**) SEM images of Au/ZIS6 at different magnifications. (**e**) SEM mapping of Au/ZIS6. (**f**–**h**) TEM images of Au/ZIS6 at different magnifications, with (**g**) showing the HRTEM image.

Figure 1a illustrates the synthesis of the Au/ZIS6 composite via a two-step process. Initially, ZIS6 was synthesized using a modified hydrothermal method, followed by the in situ photodeposition of gold (Au) nanoparticles onto the ZIS6 surface. This method leads to the formation of the Au/ZIS6 heterostructure. The SEM images in Figure 1b–d reveal the spherical morphology of the Au/ZIS6 composite, with each sphere composed of interlaced nanosheets, suggesting a hierarchical structure. The TEM image in Figure 1f provides further confirmation of this structure, demonstrating the intricate morphology of the composite. The SEM mapping in Figure 1e shows that Zn, In, S, and Au are uniformly distributed throughout the composite, confirming the homogeneous incorporation of Au into the ZIS6 matrix. At a higher magnification, Figure 1g shows that the Au nanoparticles are uniformly distributed across the nanosheet surface, ensuring efficient incorporation of Au into the ZIS6 matrix. The HRTEM image in Figure 1h displays well-defined lattice fringes, confirming the high crystallinity of the composite. Notably, the lattice spacing of 0.190 nm corresponds to the (110) plane of ZIS6, and the 0.204 nm spacing is attributed to the (200) plane of the Au nanoparticles, thus providing strong evidence for the successful formation of the Au/ZIS6 heterojunction. The size of the Au nanoparticles, as measured from the HRTEM images, is approximately 10 nm, and the above results further support the successful incorporation of Au into the composite.

### 2.2. XPS Analysis

**Figure 2:**
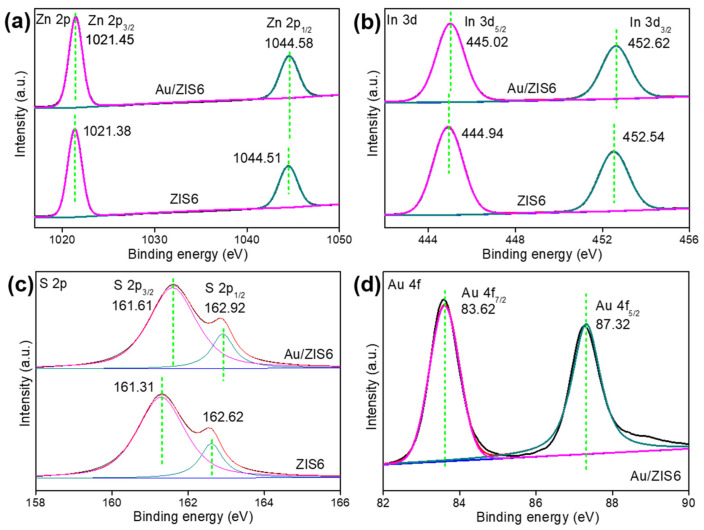
XPS spectra of ZIS6 and Au/ZIS6: (**a**) Zn 2p, (**b**) In 3d, (**c**) S 2p and (**d**) Au 4f.

The chemical states of ZIS6 and Au/ZIS6 were analyzed using XPS, with the results shown in Figure 2. In Figure 2a, the peaks at 1021.45 eV, 1021.38 eV and 1044.58 eV, 1044.51 eV correspond to the binding energies of Zn 2p_3/2_ and Zn 2p_1/2_, confirming the presence of zinc in both ZIS6 and Au/ZIS6 composites. In Figure 2b, the peaks at 445.02 eV, 444.94 eV and 452.62 eV, 452.54 eV are attributed to the In 3d_5/2_ and In 3d_3/2_ levels, indicating that indium exists as In^3+^ in both materials. In Figure 2c, the peaks at 161.61 eV, 161.31 eV and 162.92 eV, 162.62 eV correspond to the binding energies of S 2p_3/2_ and S 2p_1/2_, confirming the presence of sulfur in the composites. Finally, in Figure 2d, the XPS peaks at 83.62 eV and 87.32 eV correspond to the binding energies of Au 4f_7/2_ and Au 4f_5/2_, suggesting the presence of elemental gold in the Au/ZIS6 composite.

Furthermore, by comparing the specific peak values of the pure materials and the composites, the observed binding energy shifts further support the formation of a Schottky structure between the Au nanoparticles and the ZIS6 semiconductor. Compared to the pure materials, the Zn 2p, In 3d, and S 2p peaks in the composite shift to higher binding energies, indicating electron transfer from the semiconductor to the metal, confirming the formation of the Schottky structure in the Au/ZIS6 composite.

### 2.3. Physical Properties and Band Structure Analysis

**Figure 3:**
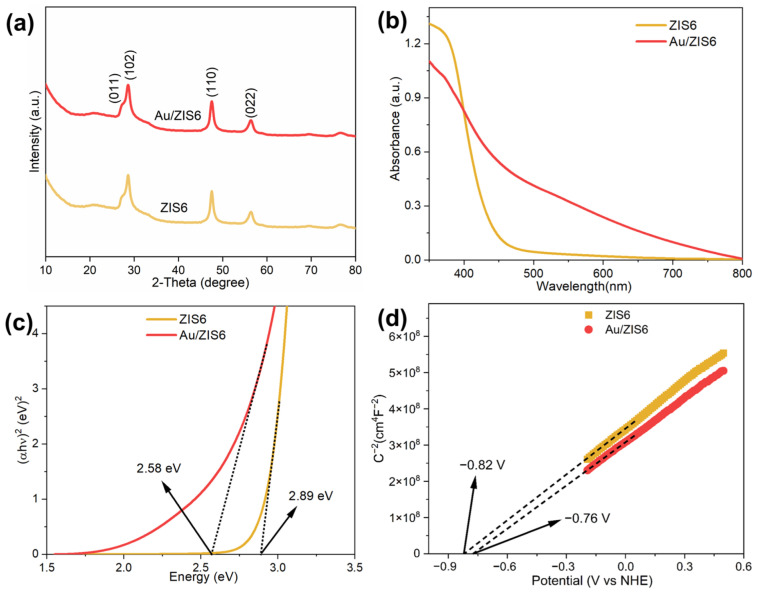
(**a**) XRD, (**b**) UV–vis DRS, (**c**) band-gap energies and (**d**) M-S plots of ZIS6 and Au/ZIS6.

Further characterization by XRD analysis (Figure 3a) reveals that ZIS6 crystallizes in the hexagonal phase of Zn_3_In_2_S_6_ (PDF#65-4003), with characteristic diffraction peaks at 26.9°, 28.2°, 46.9°, and 55.7° corresponding to the (011), (102), (110), and (022) planes of the hexagonal ZIS6 structure, respectively. This confirms the high crystallinity of ZIS6 in its pure form. Notably, no distinct peaks from Au are observed in the XRD pattern of the Au/ZIS6 composite. This absence is likely due to the uniform dispersion of Au nanoparticles at low concentrations within the ZIS6 matrix, as indicated by inductively coupled plasma (ICP) results showing the Au content in the 3% Au/ZIS6 composite to be 1.56%. These findings suggest that the Au nanoparticles are effectively integrated into the ZIS6 framework, likely forming a stable heterojunction without significantly altering the overall crystalline structure of ZIS6.

The UV–vis DRS shown in Figure 3b illustrate a significant enhancement in the light absorption of ZIS6 upon loading with Au. This is further evidenced by a decrease in the band gap from 2.89 eV to 2.58 eV after Au deposition, indicating that the Au nanoparticles help extend the absorption spectrum of ZIS6 into the visible light range. This narrowing of the band gap is advantageous for photocatalytic applications, as it allows the composite to absorb a broader range of the solar spectrum. The Mott–Schottky (M-S) plots (Figure 3d) reveal positive slopes for both ZIS6 and Au/ZIS6, confirming their n-type semiconductor behavior. The flat band potential of ZIS6 is −0.82 V, and that of Au/ZIS6 is −0.76 V (vs. normal hydrogen electrode (NHE)), with the difference being attributed to the influence of Au on the electronic structure of ZIS6. Based on the flat band potential, the conduction band positions for ZIS6 and Au/ZIS6 are calculated to be −0.92 V and −0.86 V, respectively. The valence band positions are 1.97 V for ZIS6 and 1.72 V for Au/ZIS6. These positions suggest that both ZIS6 and Au/ZIS6 are capable of generating superoxide radicals (·O_2_^−^) via the reduction of oxygen by photogenerated electrons, which plays a crucial role in the photocatalytic degradation of organic pollutants. The optimized electronic structure and enhanced light absorption indicate that Au/ZIS6 is a highly efficient photocatalyst for applications in environmental remediation and energy conversion.

### 2.4. Analysis of Photocatalytic Activity and Cycling Stability

**Figure 4:**
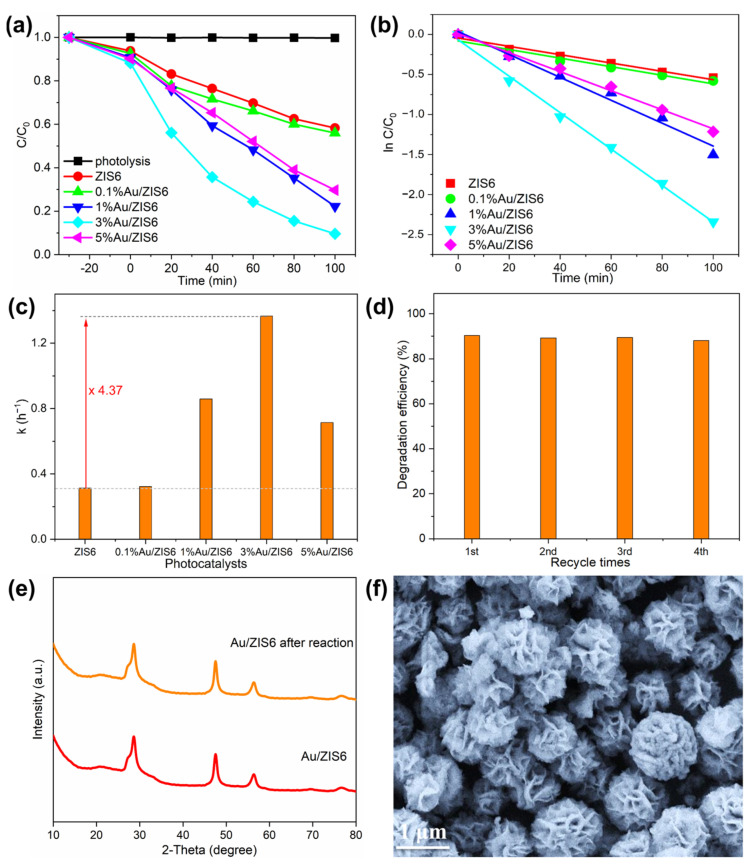
(**a**,**b**) Photocatalytic activities and (**c**) reaction rate constants of ZIS6 and Au/ZIS6 hybrids with different weight ratios of Au for photocatalytic degradation of BPA under UV–visible light. (**d**) Recycle tests of 3%Au/ZIS6 for photocatalytic degradation of BPA under UV–visible light. (**e**) XRD patterns of 3%Au/ZIS6 before and after reaction. (**f**) SEM image of 3%Au/ZIS6 after reaction.

The photocatalytic activities of ZIS6 and Au/ZIS6 composites were rigorously evaluated by their ability to degrade BPA under UV–visible light irradiation. As depicted in Figure 4a, the photolysis experiment shows that BPA does not undergo any significant degradation in the absence of a photocatalyst, confirming that the photocatalytic activity is solely attributed to the materials under investigation. Upon incorporating Au into the ZIS6 matrix, the composite’s ability to adsorb BPA increases, although the primary enhancement is observed in the photocatalytic degradation. Specifically, the 3% Au/ZIS6 composite exhibits the highest photocatalytic efficiency, achieving a remarkable 90.4% BPA degradation after 100 min of light exposure. This improvement can be attributed to the formation of a Schottky junction at the interface between Au and ZIS6. The Schottky junction not only improves the charge separation efficiency but also facilitates the rapid transfer of photogenerated electrons to the conduction band of ZIS6, thereby preventing charge recombination. Kinetic analysis (Figure 4b) reveals that the degradation of BPA follows first-order kinetics, with the first-order rate constant for 3% Au/ZIS6 being 1.366 h^−1^, which is approximately 4.37 times higher than that of pure ZIS6 (As indicated by the red arrow in Figure 4c). This significant enhancement in photocatalytic performance is consistent with the increased efficiency of charge carrier separation and the enhanced redox activity at the Au-ZIS6 interface.

Additionally, the stability of the Au/ZIS6 composite was evaluated through a series of photocatalytic cycles. As shown in Figure 4d, the photocatalytic efficiency of Au/ZIS6 remains almost unchanged after four cycles, indicating excellent cycle stability. The structural integrity and morphology of the composite were further confirmed by XRD (Figure 4e) and SEM (Figure 4f) analyses, which show no significant changes in either the crystalline phase or surface morphology after multiple photocatalytic cycles. These results suggest that the Au/ZIS6 composite not only possesses high photocatalytic activity but also exhibits outstanding stability, making it an ideal candidate for long-term applications in the degradation of persistent organic pollutants such as BPA. The incorporation of Au enhances the charge dynamics at the interface and stabilizes the overall photocatalytic system, paving the way for its potential use in large-scale environmental remediation processes.

### 2.5. Electrochemical Analysis

Enhanced light absorption and improved BPA adsorption significantly promote the photocatalytic activity of ZIS6 and its composites. However, the critical factor for boosting photocatalytic efficiency lies in inhibiting the recombination of photogenerated charges and enhancing their separation and migration. These processes are essential for improving the overall photocatalytic performance. PEC tests were carried out to examine the charge transfer and separation dynamics in the Au/ZIS6 composite.

**Figure 5:**
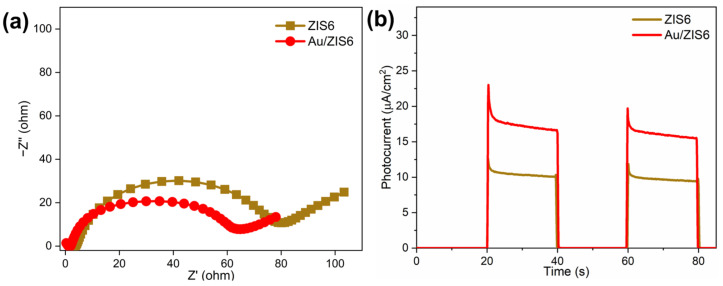
(**a**) EIS and (**b**) photocurrent spectra of ZIS6 and 3%Au/ZIS6.

As shown in Figure 5a, the impedance spectrum of Au/ZIS6 shows a smaller arc radius compared to pure ZIS6, indicating that the introduction of Au improves the conductivity of the ZIS6 semiconductor. This enhancement in conductivity facilitates faster electron transfer and contributes to the enhanced photocatalytic activity. Additionally, the photocurrent measurements (Figure 5b) reveal that the Au/ZIS6 composite exhibits significantly stronger photocurrent responses compared to ZIS6 alone. This suggests that Au nanoparticles not only improve the separation of photogenerated electrons and holes but also promote their efficient migration, thus reducing charge recombination. The enhanced charge dynamics in the Au/ZIS6 composite result in improved photocatalytic performance, confirming that Au/ZIS6 exhibits superior photocatalytic efficiency due to its ability to efficiently separate and transfer charges under light irradiation.

### 2.6. Free Radical Scavenging and ESR Analysis

**Figure 6:**
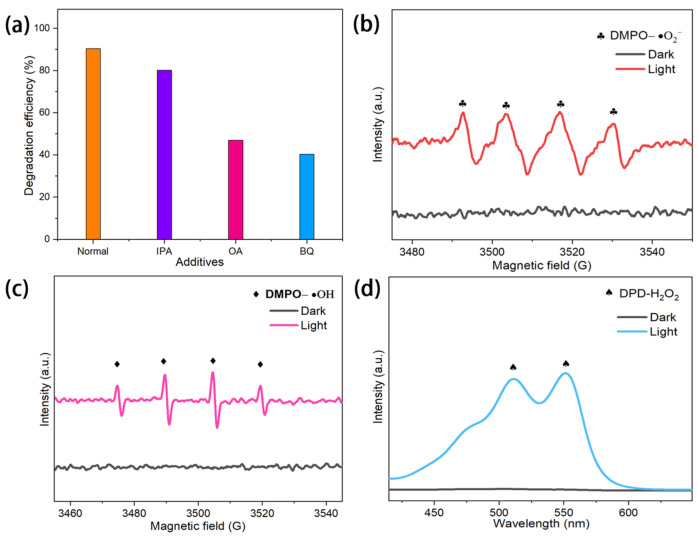
(**a**) Photocatalytic degradation efficiency of BPA with trapping agents. (**b**) ESR spectra of ·O_2_^−^ under light and dark conditions. (**c**) ESR spectra of ·OH under light and dark conditions. (**d**) H_2_O_2_ detection in the Au/ZIS6 system under light and dark conditions.

To further investigate the active species responsible for the photocatalytic degradation of BPA by Au/ZIS6, a series of trapping agent experiments were conducted. Specifically, isopropanol (IPA), ammonium oxalate (OA), and p-benzoquinone (BQ) were used as scavengers for ·OH, h^+^, and ·O_2_^−^, respectively. As shown in Figure 6a, the trapping agent experiments indicate that the addition of IPA, OA, and BQ all led to a partial reduction in the photocatalytic degradation efficiency of BPA by Au/ZIS6. The degradation efficiencies were 90.4%, 80.1%, 46.9%, and 40.3% for Normal, IPA, OA, and BQ, respectively, confirming that ·OH, h^+^, and ·O_2_^−^ are the primary active species involved in the degradation process. Among these, ·O_2_^−^ is identified as the most important species, followed by h^+^, and ·OH. This hierarchy of active species highlights the predominant role of ·O_2_^−^ in driving the photocatalytic reaction.

To verify the presence of these active species, ESR spectroscopy was employed. The ESR spectra in Figure 6b,c clearly show the formation of ·O_2_^−^ and ·OH under light irradiation, confirming their involvement in the photocatalytic process. Furthermore, the photogenerated h^+^ were detected using the diphenylpicrylhydrazyl (DPD) method, as shown in Figure 6d, indicating that Au/ZIS6 also generates h^+^ under light exposure. Additionally, the production of H_2_O_2_ in the Au/ZIS6 system, observed in Figure 6d, suggests that ·OH originates from the photolysis of H_2_O_2_. The relatively small valence band position of Au/ZIS6 (1.72 V vs. NHE) limits the ability of photogenerated holes to oxidize water into ·OH, as the oxidation potential for H_2_O/OH^−^ is 2.4 V. This indicates that H_2_O_2_ plays a crucial role in the generation of ·OH in the photocatalytic system.

### 2.7. Analysis of Charge Transfer and Catalytic Mechanisms

**Figure 7:**
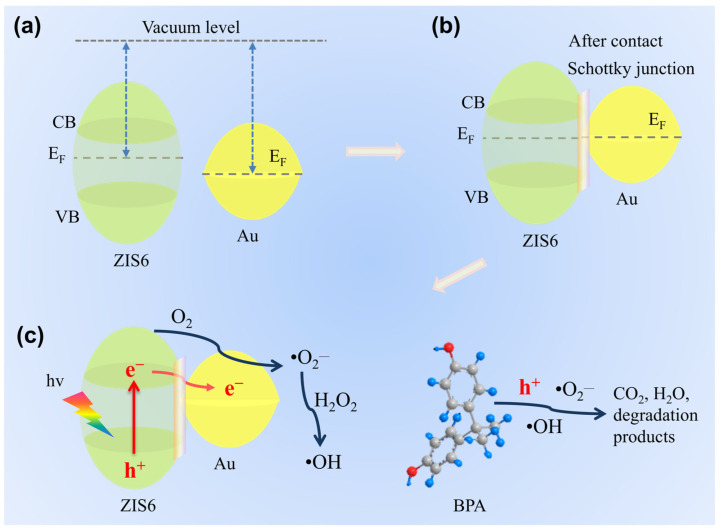
Energy level lineup diagrams for ZIS6 and Au (**a**) before and (**b**) after contact. (**c**) Schematic diagram illustrating the charge transfer and the photocatalytic mechanism for BPA degradation.

The photocatalytic degradation mechanism of BPA by Au/ZIS6 is depicted in Figure 7. When Au comes into contact with ZIS6, a Schottky junction forms at the interface due to the difference in their work functions—Au having a larger work function (5.1 eV) compared to ZIS6 (4.6 eV). This disparity leads to the downward shift in the Fermi level (E_F_) of Au relative to ZIS6, resulting in the formation of a Schottky barrier (Figure 7b). This Schottky junction is critical for enhancing photocatalytic efficiency, as it aids in the effective separation of photogenerated e^−^ and h^+^ pairs. The junction facilitates the migration of e^−^ to the Au nanoparticles, preventing their recombination with h^+^, thus significantly improving charge separation efficiency and extending the lifetime of photogenerated carriers, which are key to efficient photocatalytic processes.

Upon light irradiation, e^−^ in the conduction band (CB) of ZIS6 are excited and transferred to the Au nanoparticles, where they are captured by molecular oxygen (O_2_) to form ·O_2_^−^. These ·O_2_^−^ radicals are highly reactive and can further react with water to generate hydrogen peroxide (H_2_O_2_), which, upon decomposition, produces ·OH. The photogenerated h^+^ in the valence band (VB) of ZIS6 also play a significant role in BPA degradation. These h^+^ can directly oxidize BPA or react with O_2_ to generate more ·OH. The combined actions of h^+^, ·O_2_^−^, and ·OH lead to the breakdown of BPA, breaking the molecular bonds and ultimately converting it into environmentally benign products such as CO_2_ and H_2_O. The efficient generation and involvement of reactive oxygen species (ROS) such as ·O_2_^−^ and ·OH under visible light irradiation are crucial for the complete degradation of BPA and the overall photocatalytic performance of the Au/ZIS6 composite.

The formation of the Schottky junction also optimizes the band structure of ZIS6, making it more responsive to visible light and enabling the composite to absorb a broader spectrum of light. By facilitating charge separation and improving charge carrier dynamics, the Au/ZIS6 composite exhibits enhanced photocatalytic activity compared to pure ZIS6. This mechanism underscores the synergistic role of the Schottky junction and the generation of ROS in boosting the photocatalytic degradation of organic pollutants, paving the way for more efficient and sustainable photocatalytic systems for environmental remediation.

## 3. Materials and Methods

### 3.1. Chemicals

Gold chloride trihydrate (HAuCl_4_·3H_2_O, ≥99.9%), thioacetamide (CH_3_CSNH_2_, ≥99.0%), indium chloride tetrahydrate (InCl_3_·4H_2_O, ≥99.99%), zinc sulfate heptahydrate (ZnSO_4_·7H_2_O, ≥99.5%), hexadecyltrimethylammonium bromide (CTAB, 99%), bisphenol A (C_15_H_16_O_2_, ≥99.0%), p-benzoquinone (C_6_H_4_O_2_, ≥99.5%), ammonium oxalate monohydrate (NH_4_)_2_C_2_O_4_·H_2_O, ≥99.8%), isopropanol (IPA, 99.7%), 2,2,6,6-tetramethylpiperidine-1-oxyl (TEMPO, 97%), and 5-dimethyl-1-pyrroline-N-oxide (DMPO, 97%) were purchased from Shanghai Aladdin Biochemical Technology Co., Ltd. (Shanghai, China). All chemicals were used as received without further purification.

### 3.2. Preparation Procedures of Zn_3_In_2_S_6_ and Au/Zn_3_In_2_S_6_

The ZIS6 photocatalyst was synthesized using a modified hydrothermal method as reported [56]. Specifically, 3 mmol of ZnSO_4_·7H_2_O, 2 mmol of InCl_3_·4H_2_O, 12 mmol of CH_3_CSNH_2_, and 0.6 g of CTAB were dissolved in 30 mL of ethanol under vigorous stirring. The resulting mixture was then transferred to a Teflon-lined stainless steel autoclave and heated at 160 °C for 24 h. After the reaction, the precipitate was washed multiple times with anhydrous ethanol and deionized water, then dried in a vacuum oven at 60 °C.

For the preparation of 1% Au/ZIS6, 100 mg of ZIS6 was dispersed in 50 mL of ethanol. The HAuCl_4_ solution was then added dropwise into the suspension under vigorous stirring. The mixture was illuminated with a 300 W Xenon lamp (wavelength range 300 nm to 800 nm, MC-PF300C, Merry Change Technology Co., Ltd., Beijing, China) for 4 h. Finally, the precipitate was washed several times with anhydrous ethanol and deionized water and dried in a vacuum oven at 60 °C.

### 3.3. Characterizations

The as-prepared samples were characterized using several techniques, including XRD (Bruker D8 Advance, Karlsruhe, Germany), XPS (ESCALAB 250, VG Scientific, Hailsham, UK), field emission scanning electron microscopy (FESEM, FEI Nova Nano 230, Thermo Fisher Scientific, Hillsboro, OR, USA), TEM (FEI Tecnai G^2^-20, Hillsboro, OR, USA), and high-resolution transmission electron microscopy (HRTEM, FEI Tecnai G^2^-20, Hillsboro, OR, USA). UV–visible DRS (UV–vis DRS) were measured using a UV–vis spectrophotometer (Shimadzu UV-3600, Shimadzu Corporation, Kyoto, Japan) with BaSO_4_ (Shanghai Aladdin Biochemical Technology Co., Ltd., Shanghai, China) as the reference. Photogenerated electrons, holes, superoxide radicals, and hydroxyl radicals were detected by electron paramagnetic resonance (EPR, Bruker A300, Wissembourg, Germany). PEC tests were conducted on a CHI-660E (Chen Hua Instruments Co., Ltd., Shanghai, China) electrochemical workstation using a three-electrode system. To prepare the working electrode, a slurry was made by mixing 10 mg of the catalyst with 10 μL of 5% Nafion and 0.1 mL of ethyl alcohol. The resulting mixture was then coated onto an ITO glass substrate (1.0 cm^2^) to form the electrode. The photoelectrochemical current was recorded under simulated solar light illumination to evaluate the photocatalytic performance. A 300 W Xenon lamp (MC-PF300C, Merry Change Technology Co., Ltd., Beijing, China) with a wavelength range of 300 nm to 800 nm was used as the UV–visible light source. The distance between the light source and the sample was maintained at 10 cm during the photocatalytic measurements to ensure consistent light intensity and accurate results.

### 3.4. Photocatalytic Performance Test

The photocatalytic activity for BPA degradation was evaluated in a photocatalytic reaction system (JA-GHX1, Jingao Technology Co., Ltd., Hebei, China). In a typical experiment, 10 mg of photocatalyst and 30 mL of 10 ppm BPA solution were placed in a sealed quartz reactor. After achieving adsorption–desorption equilibrium under dark conditions for 30 min, the suspension was irradiated with UV–visible light (wavelength range 300 nm to 800 nm) from a 300 W Xe lamp. The concentration of BPA in the aqueous solution was monitored using a Shimadzu UV-3600 UV–vis-NIR spectrometer (Kyoto, Japan). The exact wavelength used for obtaining the quantitative data for analyte kinetics was 276 nm, which corresponds to the absorption peak of BPA. The photocatalytic degradation efficiency of BPA was calculated using the following formula:

Degradation efficiency = (1 − C_t_/C_0_) × 100%,

where C_0_ is the initial concentration of BPA before illumination and C_t_ is the concentration after irradiation for time t. The kinetic constant (k) of the photocatalysts was determined from the following equation:

ln (C_t_/C_0_) = −kt.

## 4. Conclusions

The Au/Zn_3_In_2_S_6_ (Au/ZIS6) Schottky junction photocatalyst exhibits exceptional photocatalytic performance for BPA degradation under UV–visible light, driven by the unique formation of a Schottky junction at the Au/ZIS6 interface. This design is a significant innovation, as it not only enhances charge separation and inhibits recombination, but also boosts light absorption and promotes efficient BPA adsorption. Gold nanoparticles play a key role in optimizing these processes, extending the photocatalytic activity into the visible light spectrum. The identification of superoxide radicals (·O_2_^−^), photogenerated holes (h^+^), and hydroxyl radicals (·OH) as the primary reactive species further highlights the mechanistic advancement in photocatalytic degradation. This work provides new insights into the design of metal-modified semiconductors, emphasizing the pivotal role of gold in advancing photocatalytic efficiency for environmental remediation.

## Data Availability

The original contributions presented in this study are included in the article. Further inquiries can be directed to the corresponding authors.
